# Adversarial Decision-Making for Moving Target Defense: A Multi-Agent Markov Game and Reinforcement Learning Approach

**DOI:** 10.3390/e25040605

**Published:** 2023-04-02

**Authors:** Qian Yao, Yongjie Wang, Xinli Xiong, Peng Wang, Yang Li

**Affiliations:** 1 College of Electronic Engineering, National University of Defense Technology, Hefei 230037, China; 2 Anhui Province Key Laboratory of Cyberspace Security Situation Awareness and Evaluation, Hefei 230037, China

**Keywords:** reinforcement learning, multi-agent Markov games, decision-making, proactive defense, moving target defense

## Abstract

Reinforcement learning has shown a great ability and has defeated human beings in the field of real-time strategy games. In recent years, reinforcement learning has been used in cyberspace to carry out automated and intelligent attacks. Traditional defense methods are not enough to deal with this problem, so it is necessary to design defense agents to counter intelligent attacks. The interaction between the attack agent and the defense agent can be modeled as a multi-agent Markov game. In this paper, an adversarial decision-making approach that combines the Bayesian Strong Stackelberg and the WoLF algorithms was proposed to obtain the equilibrium point of multi-agent Markov games. With this method, the defense agent can obtain the adversarial decision-making strategy as well as continuously adjust the strategy in cyberspace. As verified in experiments, the defense agent should attach importance to short-term rewards in the process of a real-time game between the attack agent and the defense agent. The proposed approach can obtain the largest rewards for defense agent compared with the classic Nash-Q and URS-Q algorithms. In addition, the proposed approach adjusts the action selection probability dynamically, so that the decision entropy of optimal action gradually decreases.

## 1. Introduction

In recent years, many high-level AI, such as AlphaGo, AlphaStar, Pluribus, and Suphx have defeated human players in human–machine confrontation scenarios [1], and significant breakthroughs have been made in the development of intelligent games. In terms of cyber attacks, automated and intelligent attack tools keep emerging in recent years, such as CyberBattleSim [2], NetworkAttackSimulator [3], CyberOrg [4], CyGIL [5], CALDERA [6], etc., aiming at using agents to automatically dispatch attack payloads and plan attack paths and improving the intelligence level of network attack capability. The automatic network attack platforms have greatly decreased the technical threshold of a network attack, the network attack means based on artificial intelligence technology are more concealed, and the game in cyberspace has developed to the stage of intelligent confrontation. Attackers can carry out high-intensity, high-concealment, and highly destructive attacks at a lower cost by adopting automated attack platforms and intelligent means. The situation of cybersecurity is becoming increasingly severe, the traditional defense system can no longer meet the current security requirements. Furthermore, there are inevitable defects when humans confront automatic and intelligent machines. This is because the human has physical and psychological limits, and human is more susceptible to external factors.

The similarity, deterministic, and static are the security defects of network systems, which cannot stand long-term observation, analysis, and repeated attacks. To reverse the passive situation of the network defense, the moving target defense (MTD) [7] emerges at the time required. MTD incorporates a “noise” into system and the entropy of the system configuration increases. Thus, MTD can resist the same type of attack, and greatly decreases the success rate of attacks [8]. How to efficiently use MTD technology and enhance the defense efficiency of target systems has become an important research hotspot. However, there are also vulnerabilities in MTD technology. Winterrose et al. [9] proposed adaptive attacker strategies against temporal platform migration defense. Therefore, it is urgent to study an adversarial decision-making strategy in an intelligent game scenario, to provide technical support for reversing the cyberspace security situation. Fast, automatic, and intelligent defense will be needed in the network defense field in the future. Intelligent game technology can extend the human brain and counter intelligent attacks, to improve the defense decision-making ability and adapt to the high-speed, complex, and changeable network attack and defense environment. Virtual defense agents can be used to assist defenders to make decisions.

The antagonism of goals, non-cooperation of relations, and dependence on strategies in the process of network attack and defense conform to the basic characteristics of game theory [10,11]. Confronted with intelligent attacks, network attack and defense can be modeled as a multi-agent game model, thus providing auxiliary decision support for defenders. The state transition of network attack and defense can be characterized by Markov Decision Process. Markov game regards the network attack and defense as a dynamic multi-stage process with continuous, random, and dynamic interaction.

Multi-agent reinforcement learning (MARL) [12] is an extension of reinforcement learning (RL) in a multi-agent domain. MARL is widely used in multi-agent collaboration, such as MADDPG [13], MAPPO [14], etc. However, according to the confrontation between network attack and defense, we incorporate MARL into multi-agent games. At present, some algorithms are used in multi-agent games, such as Minimax-Q [15], Nash-Q [16], Bayesian Strong Stackelberg (BSS) [17], etc. These algorithms can find the equilibrium solution of the game. However, a shared limitation of these algorithms is that they cannot dynamically adjust their strategies according to the game process. WoLF-PHC [18] can dynamically adjust the strategy; however, the performance of WoLF-PHC is not high enough to find the optimal solution of defense agent.

In conclusion, the main challenges of adversarial decision-making strategy for cyber defense are as follows:

- Challenge 1: traditional defense methods are not enough to resist automatic and intelligent attacks, so it is necessary to construct a simulation environment of multi-agent games and provide assistant decision support for defenders.
- Challenge 2: the defense strategy obtained by mainstream algorithms is certain, and it cannot adjust the strategy adaptively and continuously in the dynamic game process.
- Challenge 3: reinforcement learning algorithms used in the game should not only have both convergence and rationality, but also obtain the maximum benefit. However, it is difficult to achieve the convergence condition of the Nash-Q algorithm, and the URS-Q algorithm does not have good rationality.

In the paper, a multi-stage Markov game based on MARL is constructed, and then an optimal decision-making approach to moving target defense called WoLF-BSS-Q is proposed. The main idea of the WoLF-BSS-Q algorithm is the defense agents select the optimal defense strategy in multi-agent game and continuously and dynamically update the defense strategy by observing the environment. A series of experiments were performed to validate the efficiency and effectiveness of our proposed method. The main contributions are as follows:

- According to the characteristics of continuity, randomness, and dynamics of cyber attack and defense, the multi-agent Markov games (MAMGs) model was constructed and the equilibrium points using reinforcement learning were solved.
- The proposed WoLF-BSS-Q algorithm selects the adversarial strategy from two dimensions: micro-selection level and macro-adjustment level. Firstly, the BSS algorithm is used to select the strategy for MTD, and then the WoLF algorithm is used to dynamically adjust the strategy by observing the game process. Finally, UCB algorithm is used to choose the final strategy with the largest Q value.
- The adversarial strategy of defense agent obtained by the WoLF-BSS-Q algorithm is better than the Nash-Q and URS-Q. Furthermore, the convergence and rationality of the WoLF-BSS-Q algorithm are both excellent. From the perspective of decision entropy, the selection probability of actions can be gradually optimized using WoLF-BSS-Q.

The proposed WoLF-BSS-Q approach combines BSS, WoLF, UCB, and Constrained Q-learning algorithms. It makes use of the information intentionally or unintentionally exposed by the attacker and is expected to resist automatic and intelligent attacks based on reinforcement learning in cyberspace, which can provide assistant decision support for the defender. Meanwhile, the optimal defense strategy generated by the defense agent is allocated to each dynamic defense node, restraining and resisting the attack agent to continue to carry out high-intensity, high-concealment, and highly destruction attacks.

The rest of the paper is organized as follows. In Section 2, we review the related work of Markov games and MARL algorithms. In Section 3, we present the model of multi-agent Markov games and the main idea of our proposed WoLF-BSS-Q algorithm. In Section 4, to examine the effectiveness of WoLF-BSS-Q, a series of experiments were carried out and the experimental results were analyzed. In Section 5, we summarize the paper. All the frequent abbreviations used in our work is shown in Table A1.

## 2. Related Work

Cyberspace game has developed to the stage of intelligent confrontation. In recent years, cyber attacks have become more and more automatic and intelligent. Attackers can make full use of artificial intelligence technologies such as reinforcement learning and imitation learning to make attack decisions and deployment, and then launch high-intensity, high-concealment, and high-destructive cyber attacks with automated and intelligent attack tools. Chen et al. [19] introduce expert knowledge to guide the attack agent to make better decisions in RL-based automated attacks. Li et al. [20] construct an improved network graph model and incorporates social engineering into automated penetration testing (PT) platforms. The intelligent attack simulators [2,3,4,5,6] can construct the cyber attack model, utilize attack loads, and plan attack paths automatically, aiming at improving the intelligent level of cyber attack capability.

With the pace and complexity of cyber attacks and defense increasing, there are inevitable defects when humans confront automated and intelligent machines, mainly because of their physical and psychological limitations. Furthermore, humans are more susceptible to external factors. However, it takes a lot of time and costs to train an expert, and the successful experience of the expert is not easy to be copied widely. Therefore, it is urgent to study the adversarial decision-making approach of MTD to realize intelligent cyber defense. However, the research on proactive defense against intelligent attacks is still in its infancy. Water et al. [21] incorporate deception into CyberBattleSim for autonomous defense and deploys honeypots to confuse the attack agent. Fabio et al. [22] model PT with RL using capture-the-flag challenges and introduce port hopping into a single host. RL-based simulation environment CybORG [4] proposed the modeling methods of attackers, defenders, and arbitrators. CybORG trains in a virtual network environment and tries to run in a real network simulation environment with the same configuration through an API interface. Although the modeling of CybORG is complete, there is no focus on intelligent defense. In a word, the defense strategies against intelligent attacks remain at a static defense level, and the design of the defense algorithms is relatively simple.

Adversarial intelligent game technology is the key to realizing intelligent defense. It mainly depends on the combination of game theory and deep reinforcement learning [23]. Game theory provides an equilibrium solution to describe the learning results of multi-agent systems; however, it is mainly developed in theory and rarely applied to practical problems. Reinforcement learning provides convergent learning algorithms for the training of agents, which can achieve a rational and convergent result in the process of sequential decision-making. Defense agent uses adversarial intelligent game technology to plan the task and make real-time decisions. Markov game can be used to model an adversarial intelligent game. Furthermore, Markov game is combined with game theory and the Markov Decision Process (MDP) [15], which is suitable for describing the state transition process of a system with multi-state and stochastic characteristics. Liu et al. [24] adopts a recurrent neural network to solve the game equilibrium with partial rationality and incomplete information. Lei et al. [11] proposed an optimal strategy of MTD for the Markov game to trade off the defensive profit and network service quality.

The application of MARL into game scenarios is a new research hotspot. The agent aims to learn the reward function of each state through interaction with the environment and then learn the optimal strategy in a MARL game process. In general, it is necessary to approximately near the action-state-valued function by using the method of Q-learning. Marc et al. [25] proposed a unified game-theoretic method for MARL, they found that policies learned using independent reinforcement learning would overfit the other agents’ policies in the training process, and could not fully generalize in the implementation process. In a MARL game scenario, the agent cannot change the observation results of the opponent agent. Gleave et al. [26] believe that the observation of the opponent agent can be influenced by choosing confrontation strategies in multi-agent environments. The experiments show that the actions can naturally change the observation of opponents by training attack strategies, thus realizing the effect of black-box attacks. Aravind et al. [27] develop a game framework of model-based RL. Furthermore, they found that a near-optimal policy for the environment can be obtained by finding an approximate equilibrium for the game. Zhang et al. [28] aim to analyze the sample complexity of model-based MARL in Markov games. Xuan et al. [29] focus on the UAV swarm attack-defense confrontation based on the MADDPG algorithm.

In competitive tasks, the main purpose of MARL is how to make agents gain as many benefits as possible in competition with rivals. Littman [15] proposed the reinforcement learning algorithm Minimax-Q in a two-players zero-sum Markov game. Minimax-Q solves the minimum–maximum strategy of the game in each state for the agent to guide its action selection. Zhu et al. [30] proposed minimax Q network learning algorithm to train the network with observations and combined game theory, dynamic programming, and deep reinforcement learning to solve the Nash equilibrium for Markov games. Nash Q-learning (Nash-Q) [16] can be used in multi-player general-sum games. Nash-Q is to solve the Nash equilibrium point by quadratic programming. The convergence condition of Nash-Q is that a global optimal point or saddle point can be found in each stage game. Sailik et al. [17] proposed the Bayesian Stackelberg Markov game model and the Bayesian Strong Stackelberg Q-learning (BSS-Q) algorithm to better model the continuity and incomplete information of cyber attack and defense. The experimental results show that the BSS-Q algorithm applies to multi-agent games and is superior to Nash-Q. A shared problem of Minimax-Q, Nash-Q, and BSS-Q is that the defense agent cannot dynamically update the defense strategy by observing the environment. Policy Hill-Climbing (PHC) algorithm is a gradient descent algorithm for mixed strategies. However, the PHC strategy does not always converge. WoLF-PHC algorithm [18] is an improvement of the PHC algorithm. By incorporating the WoLF mechanism, two learning rate parameters are needed. The learning rate used for updating the strategy depends on whether the agent wins ($\delta_{w}$) or loses ($\delta_{l}$) currently. Yang et al. [31] introduce the WoLF-PHC algorithm in online RL to analyze bounded rational Markov game. Although the WoLF-PHC algorithm can update the strategy dynamically, the adjustment range is small and the performance of WoLF-PHC is not satisfactory.

In conclusion, the research on intelligent games based on MARL is booming. However, it is mainly used in real-time strategic games, UAV group operations [29], and other fields. The research on its application in the field of cyber attack and defense is still in its infancy. At present, cyber defense is still at the level of proactive defense and adaptive defense. The current cyber defense level is fragile. Therefore, it is necessary to construct a multi-agent game model of cyber attack and defense. Note that most of the existing intelligent attack platforms are based on RL algorithms. RL is easily influenced by antagonistic disturbance, which is caused by the instability of RL training. At present, the algorithms used in MARL games either cannot dynamically adjust the strategy according to the game process, or the adjustment range is too small to obtain the optimal strategy. In addition, the algorithm used in the game needs to have both convergence and rationality. Therefore, to adapt to the highly dynamic and complex cyber attack and defense environment, it can be considered to design an adversarial decision-making approach of the defense agent for MARL games.

## 3. Methodology

In this paper, a multi-agent Markov game in cyberspace according to the Cyber-Kill-Chain model is constructed. Both the attacker and the defender are agents using reinforcement learning algorithms, and the both sides can make decisions through observing the environment. Multi-agent games rely on reinforcement learning algorithms to solve game equilibrium points. Section 3.1 explains the assumptions in the method. Section 3.2 models the multi-agent Markov games. Section 3.3 introduces the proposed adversarial decision-making strategy in multi-agent Markov games, called the WoLF-BSS-Q algorithm.

### 3.1. Assumptions of Multi-Agent Markov Games

A Markov game between attack agents and defense agents in cyberspace is constructed. Furthermore, a relatively rational attacker is considered and the following assumptions are made.

- Assuming that the attack agent is rational and does not cheat or feint.
- Assuming that no third-party factors interfere with the environment.
- Assuming that the attack agent does not know that the defense agent adopts proactive defense technology and will not elaborate on the corresponding countermeasures.

### 3.2. RL Formalization for Markov Game Theory

The goal of an agent *i* is to find an optimal strategy to maximize its long-term reward in any state *s* and any time step *t* in a Markov Game.

(1) $${V_{i}}^{\pi }\left(s\right)=E_{\pi }\left\{\sum_{k=0}^{\infty }\gamma^{k}{r_{i}}^{t+k}|s_{t}=s\right\}$$

Where $V_{i}$ refers to the state-valued function of the agent *i*. *E* represents the expected value, *k* represents the future time step, γ is the discount factor, and $r_{i}^{\left(t+k\right)}$ is the reward of agent *i* at the time step (t+k). However, the state and reward function of the multi-agent environment is affected by the joint actions of all agents. Agent *i* cannot unilaterally maximize their long-term reward sum and $V_{i}\left(s\right)$. Therefore, the strategies of other agents must also be taken into account. For an agent *i*, the strategy of the agent is generally represented as $\pi_{i}:S\times A_{i}\to \left[0,1\right]$. Let the joint strategy of all agents be π = ($\pi_{1}$, $\cdots ,\pi_{n}$), the state-value function $V_{i}^{\pi }$ refers to the sum of long-term reward of agents in any state S under the joint strategy.

The action-value function $Q_{i}^{\pi }$ is the long-term reward that agent *i* can obtain when all agents in any state *S* execute joint action $\vec{a}$ and execute joint strategy π in the later process.

(2) $${Q_{i}}^{\pi }\left(s,\vec{a}\right)=E_{\pi }\left\{\sum_{k=0}^{\infty }\gamma^{k}{r_{i}}^{t+k}|s_{t}=s,\vec{a_{t}}=\vec{a}\right\}$$

To select the optimal strategy in Markov Games, the concept of equilibrium solutions in game theory has been successfully introduced into MARL, forming a lot of equilibrium-based MARL algorithms. The learning process [12] can be summarized as follows: (1) To establish a general game on the current state *S*, and taking the action value function $Q_{i}\left(s,\vec{a}\right)$ of each joint action $\vec{a}$ in this state as the reward value of agent *i* in the game of action $\vec{a}$. (2) To solve the equilibrium of the general game, and to select the action that each agent needs to perform in state *S* according to the equilibrium solution. (3) To execute the selected action and obtain the reward from the environment, and update the value function $Q_{i}$ of each agent *i* according to the reward and the equilibrium solution on the state *S*. All the joint actions of the agent *i* in state *S* are represented as $Q_{i}\left(s\right)=Q_{i}\left(s,\vec{a}|\forall \vec{a}\right)$. Furthermore, tuple $<N,{\left\{A_{i}\right\}}_{i=1}^{n},{\left\{Q_{i}\left(s\right)\right\}}_{i=1}^{n}>$ is the general game in state *S*, where $Q_{i}\left(s\right)$ corresponds to the reward function $R_{i}$ of agent *i*. Therefore, RL can be organically combined with the game theory paradigm. The mapping relationship between game theory and MARL is shown in Table 1.

**Figure 1:**
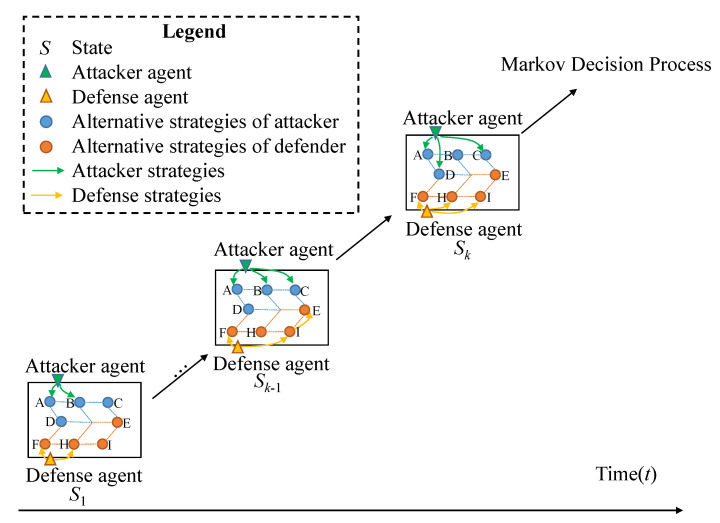
Illustration of multi-agent Markov game model.

Multi-stage Markov game is a widely used game model. It can describe the randomness, dynamics, and continuity of network attack and defense processes. In addition, Markov games can model multi-agent interactions in the sequential decision-making problem. In the game process, an agent can reason the policy from cooperative agents or opponent agents. Furthermore, the agent adopts policies based on equilibrium, where no agents can benefit by deviating from the strategies. When all participants in the game are agents, it can be called multi-agent Markov Games (MAMGs). MDP can be used to represent multi-agent Markov Games, as shown in Figure 1.

Multi-agent Markov Games can be represented by the tuple (N,S,A,τ,R,γ).

- $N=\left\{D,A\right\}$ where *D* is the defense agent and *A* is the attacker agent.
- $S=\left\{s_{1},s_{2},\cdots ,s_{k}\right\}$ are states of the game.
- $A=\left\{A^{D},A^{A}\right\}$ where $A^{D}$ and $A^{A}$ represent the action sets available to the defense agent and attacker agent in state *S*, respectively.
- τ$\left\{s,a^{D},a^{A},s^{\prime }\right\}$ denotes the probability of transferring to a state $s^{\prime }\in S$ from the state s∈S when *D* chooses $a^{D}$ and *A* chooses $a^{A}$.
- $R=\left\{R^{D},R^{A}\right\}$ where $R^{D}\left(s,a^{D},a^{A}\right)$ and $R^{A}\left(s,a^{D},a^{A}\right)$ represents the reward of defense agent and attacker agent, respectively.
- $\gamma^{i}$ is the discount factor, γ∈[0,1).

The feature of MAMGs is that the attack agent and defense agent are in the same environment, but they have their own observation space and action space. The reward value of both sides cannot be directly preset, and are influenced by the opponent’s strategy, and they obtain the reward value in the game equilibrium.

### 3.3. WoLF-BSS-Q Algorithm in Multi-Agent Markov Games

#### 3.3.1. The Basic Idea of WoLF-BSS-Q

Equilibrium-based multi-agent reinforcement learning (MARL) takes the Markov game as the learning model and the concept of equilibrium solution is introduced from game theory to define the optimal strategy. The defense agent aims to obtain an optimal strategy by interacting with the environment under the condition of incomplete information against the opponents. During the defense process, the agent interacts with the environment and it can update its incomplete information about the opponents, resulting in a Bayesian-style update after each interaction [17]. However, RL algorithms usually require a lot of interaction with real-world opponents. Therefore, we simulate a multi-agent game environment of moving target defense, where an attacker agent and a defense agent are both simulated. The defense agent selects a best strategy based on its learned knowledge, and then adjusts its strategy by observing the game process, then it can obtain a reward value when the game equilibrium is reached.

**Figure 2:**
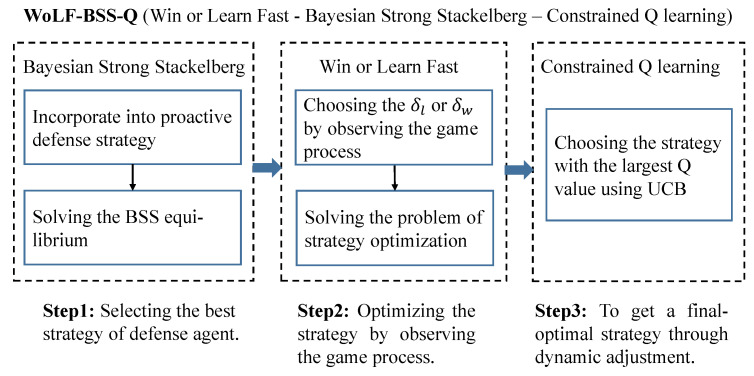
The main idea of the WoLF-BSS-Q algorithm.

The RL algorithms for MAMGs are considered to learn an adversarial decision-making strategy for the defense agents. Our proposed WoLF-BSS-Q algorithm selects the optimal strategy from two dimensions: micro-selection level and macro-dynamic adjustment level. On the one hand, given the leader-follower paradigm, the BSS algorithm can obtain the best strategy from the micro-level, on the other hand, the WoLF mechanism can dynamically adjust the parameters of the game process by observing the game process from the macro-level. By introducing the WoLF mechanism, the algorithm requires two learning rates, $\delta_{l}$ and $\delta_{w}$ ($\delta_{l}$> $\delta_{w}$).The choice of learning rate for updating the strategy depends on whether the agent wins ($\delta_{w}$) or loses ($\delta_{l}$) currently, which is decided by comparing the expected value with the current Q-value estimates. We consider introducing the WoLF mechanism to improve the performance of the BSS algorithm. Thus, the WoLF-BSS-Q algorithm is proposed for MAMGs. The main idea of the WoLF-BSS-Q algorithm is shown in Figure 2. To prevent the problem of overestimate of Q-learning, conservative Q-learning [32] is proposed. Based on the idea of this method, we proposed constrained Q-learning.

**Definition** **1.** *Constrained Q-learning is taking the average Q value as the lower limit of Q-learning to prevent the problem of overestimate of Q-learning.*

Firstly, the BSS algorithm is used to select the best strategy, and then the WoLF algorithm is used to dynamically adjust the δ value by observing the reward value of the defense agent. Because the selection strategy of ϵ-greedy is too random, leading to that some optimal actions will be assigned a very low selection probability due to initialization. To solve the problem, Upper Confidence Bound (UCB) algorithm is used to achieve the balance between the exploration and exploitation by incorporating confidence level, as shown in Equation (3). Thus, UCB algorithm is used to choose the final-optimal strategy with the largest Q value.

(3) $$A_{t}=\underset{a}{\mathrm{argmax}}\left\{Q_{t}\left(a\right)+U_{t}\left(a\right)\right\}=\underset{a}{\mathrm{argmax}}\left\{Q_{t}\left(a\right)+c\sqrt{\frac{\ln t}{N_{t}\left(a\right)}}\right\}$$

where $U_{t}\left(a\right)$ is the control coefficient of exploration intensity and determines the confidence index; *c* is a measure of uncertainty about the action; and $N_{t}\left(a\right)$ is the number of times the action is selected at time *t*.

The WoLF-BSS-Q algorithm is shown as Algorithm 1. In line 4, the unite probability of selecting an action in a certain state *S* is determined. In line 6, the scenario is modeled as a mixed-integer linear programming (MILP). Through interaction with the environment, in line 7 to line 10, the Q-values for the defense agent and the attacker agent in the state *S* are updated. Line 7 to line 9 is the constrained Q-learning algorithm. Line 11 to line 22 is the optimization process of the WoLF algorithm, $\pi (s,a^{d})$ is the optimization probability of the WoLF algorithm. In line 23, a Markov games solver is used to calculate the bi-matrix game in the state *S* of the WoLF-BSS-Q. The UCB algorithm is adopted to find the final adversarial decision-making strategy. Then, the strategy of the defense agent is optimized and updated when a new episode is started in line 4.

#### 3.3.2. The Convergence and Rationality of WoLF-BSS-Q

There are two expected properties [18] for a learning agent when there are other learning agents, namely convergence and rationality.

- **Convergence:** Convergence means that the current agent can learn and converge to a stable strategy when other agents also use learning algorithms. The BSS-Q algorithm can converge to a strong Stackelberg equilibrium (SSE) [17] of a MAMGs. By introducing the WoLF mechanism, the WoLF-BSS-Q algorithm is an improvement of the BSS-Q algorithm and has good convergence.
- **Rationality:** When the opponent uses a constant strategy, the current agent can learn and converge to an optimal strategy relative to the opponent’s strategy. The WoLF-BSS-Q algorithm has good rationality. First, the BSS-Q algorithm is used to select the best strategy, and then the WoLF algorithm is used to optimize the strategy. Through a two-step scheme, the final-optimal strategy with convergence can be chosen.

**Algorithm 1:** WoLF-BSS-Q in MAMGs

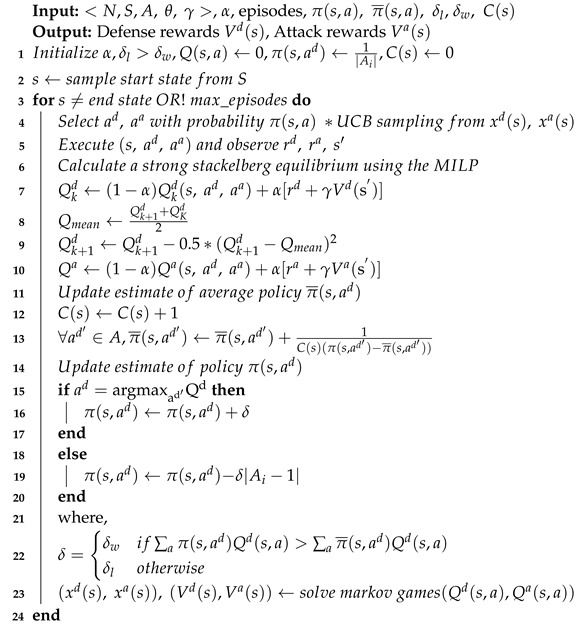



## 4. Experiments

The experiments were conducted on an Open-AI gym style multi-agent game simulator [17]. Section 4.1 describes the experimental scenario. Then we carried out a series of experiments. Section 4.2 analyzes the experimental results.

### 4.1. Application Example

**Figure 3:**
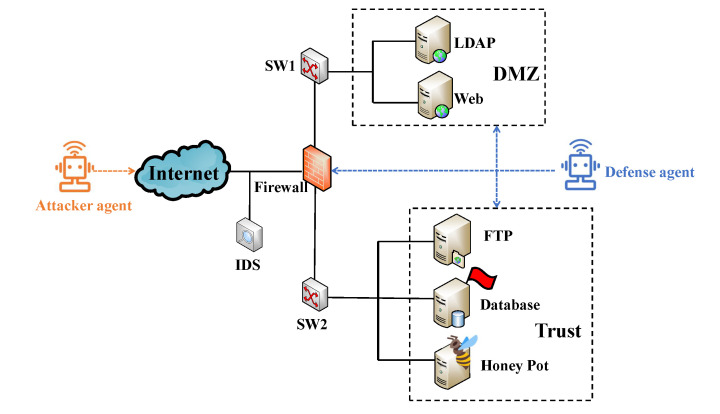
Experimental topology.

The application example is used to validate the effectiveness of the designed optimal strategy selection algorithm WoLF-BSS-Q. The network scenario consists of a DMZ zone and a Trust zone. There are hosts $h_{1},h_{2},\cdots ,h_{5}$ to provide LDAP, FTP, web and database services, respectively, as shown in Figure 3.

The attacker agent aims to obtain the sensitive data in the database. The vulnerability information of the network topology is shown in Table 2.

The whole network attack and defense process is divided into seven states $\left\{S_{1},S_{2},\cdots ,S_{7}\right\}$, $S_{7}$ is the end state, and the other states are the attack and defense process. In each state, the attack agent and defense agent both have a set of actions to select from. $A_{i}$ and $D_{i}$ represent the optional action of the attack agent and defense agent in the state $S_{i}$, respectively. The description of the attacker agent strategy is shown in Table 3. The attacker agent performs the attack action according to the Cyber-Kill-Chain model. The attacker agent exploits the CVE vulnerability by using payloads prepared in advance. The defense agent adopts traditional defense strategies and proactive defense strategies. The description of the defense agent strategy is shown in Table 4.The traditional strategies include deploying the IDS and firewall. The active strategies include honeypot deploying, IP hopping, port hopping, topology shuffling, etc. A honeypot is a replica of the database to provide some common services, open more sensitive ports, and contain some invalid resources and information. Honeypot aims to consume the attacker’s resources and mitigate the impact of the attacker’s actions on the network. Port hopping refers to the mutation of the port IDs and does not match the corresponding service on the prerequisite of not affecting the user experience, thus invalidating the information collected by the attack agent. The principle of IP hopping is similar. Network topology shuffling refers to the dynamic change of network topology, which is used to resist the horizontal penetration attack of an intranet.

### 4.2. Analysis of Experimental Results

To validate the effectiveness of WoLF-BSS-Q algorithm, we adopt a series of experiments. Firstly, Section 4.2.1 selects the most suitable parameters of WoLF-BSS-Q algorithm. Secondly, Section 4.2.2 compares with other classical algorithms. Finally, Section 4.2.3 analyzes the decision entropy of defense agent strategy.

#### 4.2.1. Comparison Results of Arameter Testing

**Figure 4:**
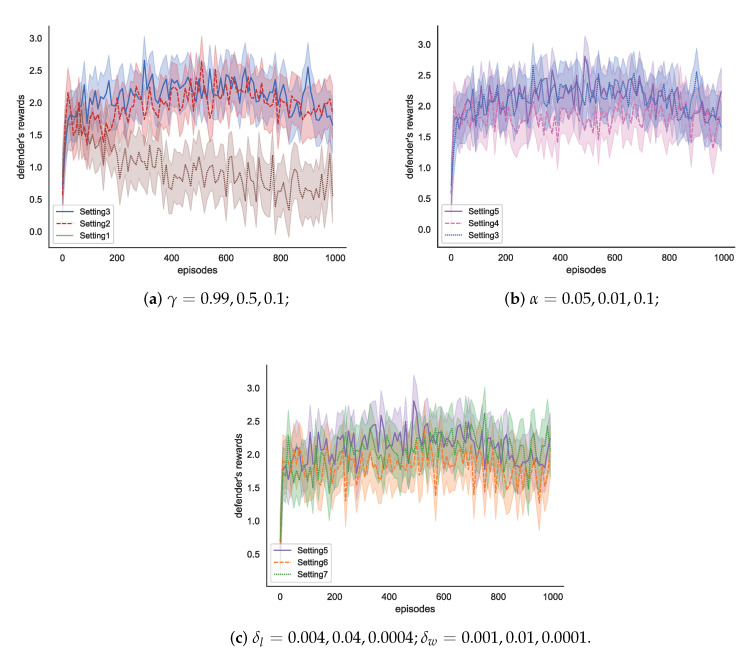
Comparison results of defense agent rewards with different parameter settings.

In this experiment, the number of episodes is 1000, the number of steps per episode is 100, and the number of trials is 100. In addition, the different hyper-parameter settings of WoLF-BSS-Q algorithm are shown in Table 5. The Setting 1 to Setting 3 is to compare the influence of γ, as shown in Figure 4a. The Setting 3 to Setting 5 is to compare the influence of α, as shown in Figure 4b. Furthermore, the Setting 5 to Setting 7 is to compare the influence of $\delta_{l}$ and $\delta_{w}$, according to the paper [18], it is appropriate when the $\delta_{l}$ is four times of the $\delta_{w}$, as shown in Figure 4c.

Combining the experimental results of Figure 4a–c, we can find that the defense agent can obtain the highest reward value with Setting 5. In this case, the parameters are γ = 0.1, α = 0.1, $\delta_{l}$ = 0.04, and $\delta_{w}$ = 0.01. γ = 0.1 means that in the game process of multi-agents, the defense agent should choose strategies according to the real-time game situation. Due to the influence of the defender’s dynamic strategy and the attacker’s behavior, the entropy value of the game environment is constantly changing. In the real-time attack and defense process, the defense agent will find the approximate optimal solution instead of the optimal solution. Therefore, if the defense agent pays too much attention to long-term rewards, it may not evaluate the current strategies accurately. Therefore, the defense agent should attach importance to short-term rewards in the process of a real-time game between the attack agent and the defense agent.

**Figure 5:**
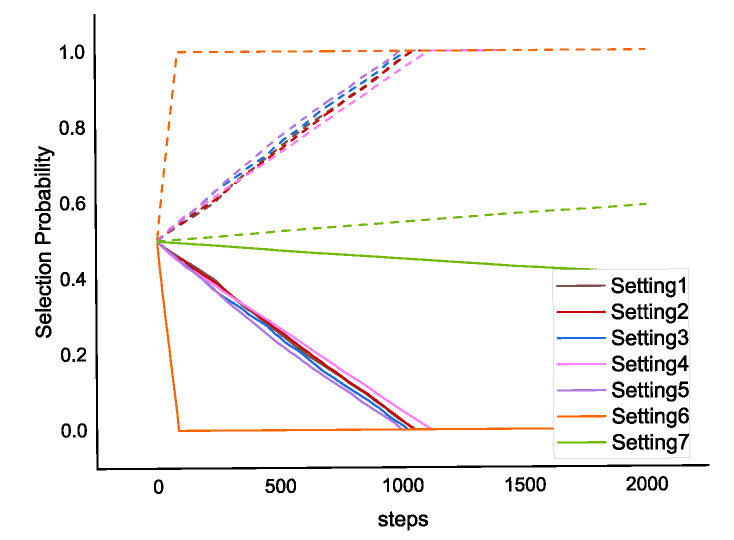
Comparison results of action selection probability of defense agent with different parameter settings.

Figure 5 is the comparison results of the action selection probability of a defense agent with different parameter settings, the parameter settings are the same as those in Table 5.

Figure 5 shows that the WoLF algorithm can dynamically adjust the selection probability of strategies in a state *S* according to observing the game process. In the state *S*, the probability of selecting one action gradually decreases, represented by solid lines. The probability of selecting another action gradually increases, represented by dotted lines. Furthermore, the sum of the selection probabilities of all actions is 1. This is also the advantage of the WoLF-BSS-Q algorithm over the BSS algorithm. When an action is more beneficial to a defense agent, the probability of being selected gradually increases; on the contrary, the probability of being selected gradually decreases. The value of δ can control the speed of parameter adjustment. Combining the experimental results of Figure 4 and Figure 5, the effectiveness of Setting5 is the best.

#### 4.2.2. Comparison with the Classical Algorithms

In this section, we compare the performance of the WoLF-BSS-Q algorithm with two baseline algorithms: (1) Nash-Q, and (2) URS-Q.

- **Nash-Q:** Nash equilibrium is the optimal solution of the Nash Q-Learning (Nash-Q) when all agents are entirely rational. Any change of unilateral strategy will bring the loss of utility value to the agent in a Nash equilibrium. Nash-Q uses quadratic programming to solve Nash equilibrium points. The convergence condition is that a Nash equilibrium point or a global optimal point can be solved in every state *S* of the game. However, Nash-Q can not always converge every time and the calculation speed of Nash-Q is slow. Therefore, Nash-Q has good rationality but does not have good convergence.
- **URS-Q:** The uniform random strategy (URS) selects each action with equal probability in each state, and will not adjust or optimize its strategy according to the opponent’s strategy. URS-Q uses Q-learning to obtain the maximum reward of the strategy. From the perspective of decision entropy, the URS-Q algorithm cannot determine which strategy is better, and the probabilities are all equally distributed. The decision entropy of the URS-Q algorithm always equals 1. In conclusion, URS-Q has good convergence but does not have good rationality.

**Figure 6:**
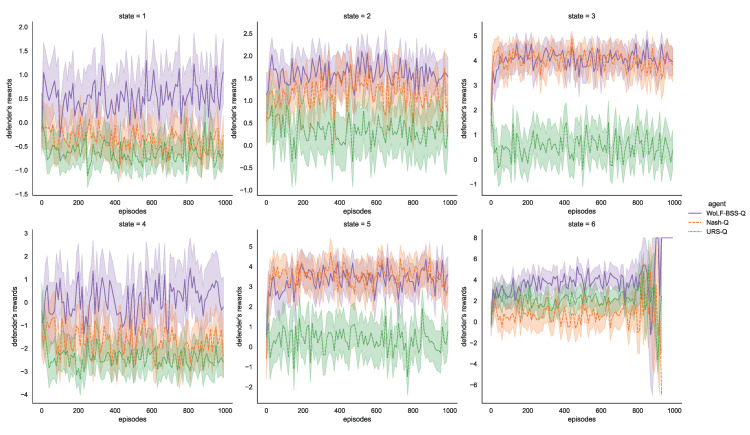
Comparison with the classical algorithms.

Based on the experiment of parameter testing, we chose Setting5 for this experiment. The hyperparameters of the three algorithms are shown in Table 6:

The comparison with the classical algorithms is shown in Figure 6. There are six states of multi-agent games in total, and the strategies adopted by the attack and defense agents are shown in Table 3 and Table 4. The reward calculation method of a multi-agent game is different from that of a single agent, which cannot be obtained by preset values, and the equilibrium solution needs to be obtained according to the opponent’s strategy. The calculation of the reward of the defense agent in our proposed method is divided into the following steps: firstly, according to the BSS algorithm, the solution of the MILP model is calculated as the action selection probability; secondly, the action selection probability is adjusted and optimized by WoLF algorithm; finally, the reward value is obtained according to UCB algorithm. Because both the attacker agent and defense agent are learning from the game process and have adversarial strategies, they may take turns to gain the upper hand. Therefore, it can be seen that the reward value of the defense agent is unstable. It may be possible to limit the training time and end the episodes artificially. According to the game process of each state, the performance of the WoLF-BSS-Q algorithm is the optimum, and it can converge to the maximal rewards of the defense agent. The performance of the Nash-Q is second. Furthermore, in state 3 and state 5, the performance of the WoLF-BSS-Q and Nash-Q is almost equal. However, the convergence of the Nash-Q is relatively poor, leading to the training results of Nash-Q cannot always be obtained. In addition, the performance of the URS-Q is the worst. This is because the probability of selecting each action in a state is equal, and URS-Q will not optimize its own strategy, as a result, URS-Q obtains the worst performance in most cases.

#### 4.2.3. The Analysis of Decision Entropy

Decision entropy is a metric for measuring the uncertainty of the action selection in a certain state, as shown in Equation (2).

(4) $$H\left(x\right)=-\sum_{i=1}^{n}p\left(x_{i}\right)logp\left(x_{i}\right)$$

The smaller the decision entropy is, the more certain the action selection probability is. It means that the action selection probability approaches to 0 or 1 and the decision entropy approaches to 0. Furthermore, the larger the decision entropy is, the more uncertain the action selection probability is. It means that the action selection probability approaches to the average value and the decision entropy approaches to 1.

Therefore, it is meaningless that the action selection probability approaches the average value, and it cannot help the defense agent to decide on an action with a higher reward value, just like the URS-Q algorithm. The Nash-Q algorithm does not involve the analysis of decision entropy. It solves the optimal strategy according to the classic Nash equilibrium, but this condition is too harsh, it cannot always find the Nash equilibrium point, and the solution time is too long. From the demand of real-time decision-making of network attack and defense, Nash equilibrium is not suitable for this scenario. WoLF-BSS-Q algorithm adjusts the action selection probability by observing the game process so that the action with greater rewards can be selected with higher probability, and thus the decision entropy gradually decreases. From the perspective of decision entropy, the WoLF-BSS-Q algorithm can give defense agents a better choice of strategies.

## 5. Conclusions and Future Work

An adversarial decision-making approach to moving target defense called WoLF-BSS-Q was proposed in this paper for multi-agent Markov games. The main idea of this method is: firstly, the BSS algorithm is used to select the defense strategy, and then the WoLF algorithm is used to dynamically adjust the learning rate by observing the rewards of the defense agent. Finally, UCB algorithm is used to choose the final adversarial decision-making strategy with the largest Q value. To prevent overestimate of Q-learning, we adopt constrained Q-learning to calculate the Q value. With this method, the defense agent can obtain the optimal strategy as well as continuously adjust the strategy. The experiments showed that the defense agent should attach importance to short-term rewards in the process of a real-time game between the attack agent and the defense agent. The WoLF-BSS-Q can obtain the highest rewards for defense agent compared with the Nash-Q and URS-Q algorithms. From the perspective of decision entropy, when the selection probability of each action is more equal, the greater the decision entropy value is. Furthermore, it has less effect on the defense agent’s decision-making. Furthermore, the proposed WoLF-BSS-Q approach adjusts the action selection probability by observing the game process, the optimal action can be selected with higher probability, and thus the decision entropy gradually decreases.

In this paper, the attack agent will not cheat to consume defense resources, nor can it identify the dynamic defense strategy of the defense agent; that is, the “intelligence level” is not high enough. In the future, we will study an irrational opponent, who can make misleading deceptions or feint. It is also worth studying a multi-agent Markov game with incomplete information, which will cause deviation in the observation space of the defense agents.

## Figures and Tables

**Table 1:** The mapping relationship between game theory and MARL.

Reinforcement Learning	Game Theory
Environment	Game
Attacker agent and Defense agent	Player1 and Player2
Action	Action
Policy	Strategy
Reward	Payoff

**Table 2:** Network topology vulnerabilities.

Server ID	CVE ID	Vulnerability Information
LDAP	CVE-2020-35949	Uploading arbitrary files and achieve remote code execution.
Web	CVE-2021-25646	Executing code on the target machine with the privileges of the Druid server process.
FTP	CVE-2019-3396	Achieving path traversal and remote code execution.
Database	CVE-2022-0847	To write to pages in the page cache backed by read only files and as such escalate their privileges on the system.
Honey Pot	CVE-2021-4034	Local privilege escalation and executing arbitrary code.

**Table 3:** The strategies of the attacker agent.

No.	Attack Strategy
A${}_{1}$	no-operation, scan
A${}_{2}$	exploit credentials, exploit vulport
A${}_{3}$	exploit CVE-2020-35949, exploit CVE-2021-25646, exploit other CVEs
A${}_{4}$	no-operation, upload trojan
A${}_{5}$	exploit CVE-2019-3396, exploit CVE-2022-0847, exploit CVE-2021-4034, exploit other CVEs
A${}_{6}$	exploit database, exploit honeypot

**Table 4:** The strategies of the defense agent.

No.	MTD Strategy
D${}_{1}$	no-monitor, IDS
D${}_{2}$	identification, port hopping
D${}_{3}$	monitor CVE-2020-35949, monitor CVE-2021-25646, IP hopping
D${}_{4}$	no-monitor, monitor trojan
D${}_{5}$	monitor CVE-2019-3396, monitor CVE-2022-0847, monitor CVE-2021-4034, topology shuffling
D${}_{6}$	monitor database, monitor honeypot

**Table 5:** The different setting of hyperparameters.

Setting Number	α	$\delta_{l}$	$\delta_{w}$	γ
Setting1	0.05	0.004	0.001	0.99
Setting2	0.05	0.004	0.001	0.5
Setting3	0.05	0.004	0.001	0.1
Setting4	0.01	0.004	0.001	0.1
Setting5	0.1	0.004	0.001	0.1
Setting6	0.1	0.04	0.01	0.1
Setting7	0.1	0.0004	0.0001	0.1

α is the learning rate, to determine how much the model parameters are adjusted in each step. δ is the adjustment of the learning rate, an offset. γ is the discount factor, to decide whether the agent focuses on long-term or short-term interests.

**Table 6:** The hyperparameters of the three algorithms.

Hyperparameters	WoLF-BSS-Q	Nash-Q	URS-Q
α	0.1	0.1	0.1
ϵ	0.1	0.1	0.1
γ	0.1	0.1	0.1
$\delta_{l}$	0.04	-	-
$\delta_{w}$	0.01	-	-

- indicates that there are no values.

## Data Availability

Not applicable.

## Appendix A

**Table A1:** Abbreviations and their descriptions.

Abbreviations	Descriptions
MARL	Multi-agent Reinforcement Learning
MAMGs	Multi-agent Markov Games
MDP	Markov Decision Process
PT	Penetration Testing
MTD	Moving Target Defense
WoLF	The Win or Learn Fast algorithm
BSS	The Bayesian Strong Stackelberg algorithm
SSE	The Strong Stackelberg Equilibrium
MILP	The Mixed Integer Linear Programming model
UCB	The Upper Confidence Bound algorithm
URS	The Uniform Random Strategy
